# Two case reports of the successful eradication of new isolates of *Burkholderia cepacia complex* in children with cystic fibrosis 

**DOI:** 10.1186/s40360-016-0054-0

**Published:** 2016-03-28

**Authors:** H. Kitt, W. Lenney, F. J. Gilchrist

**Affiliations:** Royal Stoke University Hospital, Stoke on Trent, ST4 6QG UK; Academic Department of Child Health, Royal Stoke University Hospital, Newcastle Road, Stoke on Trent, ST4 6QG UK

**Keywords:** Burkholderia cepacia complex, Cystic fibrosis, Eradication, Paediatrics

## Abstract

**Background:**

Chronic infection with Burkholderia cepacia complex (BCC) has a detrimental effect on morbidity and mortality for patients with cystic fibrosis (CF). It is therefore logical to attempt to eradicate new isolates however there is a paucity of information to guide treatment. We report the successful eradication of new isolates of BCC in two children with CF.

**Case presentation:**

*Burkholderia cepacia* was successfully eradicated in a 14 year old boy with CF and *Burkholderia gladioli* was successfully eradicated in a six year old girl with CF. In both children two weeks of intravenous (IV) tobramycin, ceftazidime and temocillin were used followed by three months of inhaled tobramycin. Bronchoalveolar lavage samples taken during flexible bronchoscopy were used prior to treatment to exclude spontaneous clearance as well as after treatment to confirm eradication.

**Conclusions:**

New isolates of BCC can be successfully eradicated in children with CF. More research is urgently required in this area to identify the best treatment regimen for BCC eradication.

## Background

Cystic fibrosis (CF) is the commonest life shortening, inherited disease in the Caucasian population. It is characterised by recurrent lower respiratory infections, inflammation and lung damage. *Burkholderia cepacia complex* (BCC) refers to a group of at least 17 closely related bacterial species (formerly called genomovars) that can cause pulmonary infection in patients with cystic fibrosis (CF) [1–3]. These Gram-negative organisms are inherently resistant to Colistin and can survive for prolonged periods in moist environments such as water and soil [1]. In patients with CF, new BCC infection can clear spontaneously or require eradication with a combination of antibiotics. If new infection is not cleared, chronic infection develops. Chronic BCC infection is associated with a more rapid decline in lung function [4], increased requirements for intravenous antibiotics [5], increased outpatient attendance and increased mortality [6]. Patient to patient spread of epidemic strains of *Burkholderia cenocepacia* (such as ET12) was identified at a number of CF centres in the early 1990’s [7]. This had a catastrophic effect on the CF population due to its association with Cepacia Syndrome. This is an acute, necrotising pneumonia with an almost universal fatal outcome [8]. The presence of *Burkholderia cenocepacia* remains a contraindication to lung transplantation at most CF transplantation centres due its association with poor outcomes [9]. The widespread segregation of CF clinics has been successful in reducing the overall prevalence of chronic BCC infection in the UK to 3 % [10]. It is predominantly seen in adult patients [10]. The success of segregation means that most new BCC infections are now acquired from the environment rather than from other patients [11].

Despite a wealth of knowledge on the adverse long-term effects of BCC infection, there is lack of reliable evidence on which to base management decisions for CF patients with new or chronic BCC infection [12, 13]. An in-vitro study suggest that triple-antibiotic combinations are more likely than double and single antibiotic combinations to be bactericidal against BCC [14]. Despite this, a Cochrane Systematic Review failed to identify a single randomised study investigating BCC eradication and concluded that “without further comprehensive studies it is difficult to draw conclusions about a safe and effective management strategy for BCC eradication in CF” [13]. A review of practice in UK CF Centres revealed that despite this lack of evidence, most attempt to eradicate new isolates of BCC. The treatment regimens vary widely and reported success was limited (37 %) [15]. The published evidence is limited to a few small case series. Some of these use a combination of intravenous and nebulised antibiotics [16], others use a combination of nebulised antibiotics [17] and others a single nebulised antibiotic [18]. We report two children in whom new isolates of BCC have been successfully eradicated using the same combination of intravenous (IV) and inhaled antibiotics. These are the only two cases of attempted BCC eradication at our centre in the last eight years.

## Case presentations

### Child 1

A 14 year old boy with CF (homozygous Phe508del) presented with a 2 week history of a wet cough productive of green sputum. His lung function remained stable (FEV_1_ 90 %) and clinical examination was unremarkable. He was known to be chronically infected with *Staphylococcus aureus* but was free from *Pseudomonas aeruginosa* (last isolate 34 months previously) and had never grown BCC. His treatment included nebulised dornase alfa but no nebulised antibiotics. The sputum culture from this presentation isolated BCC, *Staphylococcus aureus, and Haemophilus influenza*. Table 1 shows the antibiotic sensitivities. The BCC isolate was sent to the National Reference Laboratory where recA sequencing confirmed its identity as *Burkholderia cepacia*. The antibiotic sensitivities are given in Table 1. Five weeks after the first isolate, flexible bronchoscopy with single aliquot bronchoalveolar lavage samples (FB-BAL) from six lobes (including the lingula lobe) was performed to see if the organism had spontaneously cleared. BCC was isolated from all the sampled lobes. A 2 week eradication regimen was planned using intravenous (IV) tobramycin (10 mg/kg once daily), ceftazidime (50 mg/kg three times daily), and temocillin (2 g twice daily) followed by 3 months of nebulised meropenem (250 mg twice daily). The IV antibiotics were administered in hospital without any problems. Trough serum tobramycin concentrations were <1 mg/L when measured on day 2 and day 8 of treatment. Renal and liver functions were also within normal limits. Unfortunately, the patient could not tolerate the nebulised meropenem due to the taste. He was therefore prescribed 3 months of inhaled tobramycin. The possibility of using either a nebulised or a dry powder (TIP) formulation was discussed with the patient who chose the latter. The patient passed his trial of TIP and treatment was commenced (112 mg twice daily) the day after his IV antibiotics had finished. The family reported excellent adherence with the 3 months of treatment. All subsequent microbiology samples have been negative for BCC. These have included four sputum cultures whilst still on TIP, FB-BAL samples taken 1 month after the end of the eradication course and three subsequent cough swabs. The last sample was 13 months after the first BCC isolate.

**Table 1:** Antibiotic sensitivities of the two BCC isolates

	Ceftaz	Tazocin	Cipro	Gent	Tobra	Amikacin	Mero	Colistin	Aztreo	Temo
Child 1	S	S	I	R	R	R	S	R	I	S
Child 2	S	R	I	I	I	R	S	R	R	S

*Ceftaz* ceftazidime; *Cipro* ciprofloxacin; *Gent* gentamicin; *Tobra* tobramycin; *Mero* meropenem; *Aztreo* aztreonam; *Temo* temocillin; *S* sensitive; *I* intermediate; *R* resistant

### Child 2

Four weeks after the above child first isolated BCC, a 6 year old girl with CF (homozygous Phe508del) also isolated BCC from a cough swab after a four week history of cough and shortness of breath. She had isolated *Pseudomonas aeruginosa* on two previous occasions (latest 20 months ago) and was receiving nebulised dornase alpha but not nebulised antibiotics. The BCC isolate was sent to the National Reference Laboratory where gryB cluster analysis confirmed its identity as *Burkholderia gladioli*. The antibiotic sensitivities are given in Table 1. FB-BAL was performed 4 weeks after the positive culture to check if it had spontaneously cleared but BCC was isolated from all six sampled lobes. She completed 2 weeks of IV tobramycin (10 mg/kg once daily), ceftazadime (50 mg/kg three times daily) and temocillin (1 g twice daily). Trough serum tobramycin concentrations were <1 mg/L when measured on day 2 and day 8 of treatment. Renal and liver function were also within normal limits. As she had previously struggled to tolerate the taste of nebulised treatments we did not try nebulised meropenem and went straight for 3 months of nebulised tobramycin (300 mg twice daily). This was started on the day the IV antibiotics finished. She tolerated this well and the family reported excellent adherence. During this time her lung function and clinical status remained stable. All subsequent microbiology samples have been negative for BCC. This has included four cough swabs cultures whilst still on nebulised tobramycin, FB-BAL samples taken 1 month after the eradication course finished and six subsequent cough swabs. The last sample was 12 months post first BCC isolate.

## Discussion

We report two children with CF in whom new isolates of BCC were successfully eradicated using the same combination of intravenous and inhaled antibiotics. There is a paucity of evidence to inform practice on the eradication of BCC and we believe that these are the first two isolates successfully treated and reported in children. A Cochrane review concluded that there is “an extreme lack of evidence in this area of treatment management for people with cystic fibrosis” [13]. Although this report adds to the available evidence there is still an urgent need for multi-centre randomised controlled trials to investigate antibiotic regimens for the eradication of BCC.

The BCC isolates in these children were identified as *Burkholderia cepacia* and *Burkholderia gladioli*. The progression of *Burkholderia cepacia* infection and its detrimental effect on morbidity and mortality is well established [11]. The clinical significance of *Burkholderia gladioli* infection in patients with CF is slightly more controversial as infections can be transient and prevalence of chronic infection is relatively low [19]. However, there is evidence that if chronic infection does develop there is associated morbidity both pre and post-transplant [20, 21]. In these children we checked for spontaneous clearance by performing FB-BAL four/five weeks after the initial isolate. In both, BCC was identified in all six of the sampled lobes excluding this possibility. We were equally thorough in our assessment of the success of eradication. This included FB-BAL one month after eradication had completed in addition to regular respiratory microbiology samples during and after treatment.

## Conclusions

In conclusion, new isolates of BCC can be eradicated in children with CF. This report identifies one possible eradication regimen using three IV antibiotics and one nebulised antibiotic. More research is required to establish which eradication regimen is the best for new isolates of BCC.

## Ethics and informed consent

Ethical approval was not required for this article as case reports are not research. Written informed consent was obtained from the parents for publication of these case reports. A copy of the written consent is available for review by the Editor of this journal.

## Abbreviations

BCC: *Burkholderia cepacia complex*
CF: cystic fibrosis
FB-BAL: flexible bronchoscopy with bronchoalveolar lavage
IV: intravenous
TIP: tobramycin inhalation powder
